# Markov Chain Realization of Joint Integrated Probabilistic Data Association

**DOI:** 10.3390/s17122865

**Published:** 2017-12-10

**Authors:** Eui Hyuk Lee, Qian Zhang, Taek Lyul Song

**Affiliations:** 1 5th Development Division, Agency for Defense Development, P.O.Box 35, Daejeon, Korea; jobdavid@add.re.kr; 2 Department of Electronic Systems Engineering, Hanyang University, Ansan, 15588, Korea; zq2013750509@hanyang.ac.kr

**Keywords:** Markov chain data association, JIPDA, target existence, multi-target tracking

## Abstract

A practical probabilistic data association filter is proposed for tracking multiple targets in clutter. The number of joint data association events increases combinatorially with the number of measurements and the number of targets, which may become computationally impractical for even small numbers of closely located targets in real target-tracking applications in heavily cluttered environments. In this paper, a Markov chain model is proposed to generate a set of feasible joint events (FJEs) for multiple target tracking that is used to approximate the multi-target data association probabilities and the probabilities of target existence of joint integrated probabilistic data association (JIPDA). A Markov chain with the transition probabilities obtained from the integrated probabilistic data association (IPDA) for single-target tracking is designed to generate a random sequence composed of the predetermined number of FJEs without incurring additional computational cost. The FJEs generated are adjusted for the multi-target tracking environment. A computationally tractable set of these random sequences is utilized to evaluate the track-to-measurement association probabilities such that the computational burden is substantially reduced compared to the JIPDA algorithm. By a series of simulations, the track confirmation rates and target retention statistics of the proposed algorithm are compared with the other existing algorithms including JIPDA to show the effectiveness of the proposed algorithm.

## 1. Introduction

Multi-target tracking [1,2,3,4,5] is an important task of radar, sonar, acoustic, electro-optical, and infrared systems and various other tracking applications. The measurements obtained by sensors may be originated from real targets and clutter. In a multi-target environment, each target is detected with a certain probability of detection, the number of targets is unknown, and a random number of false alarms or clutter measurements are generated at the random locations in the surveillance region. Under these situations, true as well as false tracks are initiated, and a track quality measure is needed for false track discrimination (FTD) and track maintenance.

A well-known algorithm for multi-target tracking in cluttered environments is joint probabilistic data association (JPDA) [6,7], which extends probabilistic data association (PDA) for single-target tracking in clutter with a known number of targets in a cluttered environment to multi-target tracking environments. The JPDA algorithm is used to compute the track-to-measurement association probabilities for all the feasible joint events (FJEs), and the complexity of the calculation grows combinatorially with the number of targets and the number of measurements. In addition, these JPDA and PDA approaches do not have measures for discriminating false or true tracks. FTD involves confirming the tracks that follow true targets and terminating false tracks. The probability of target existence (PTE) is used as a track quality measure for FTD in integrated probabilistic data association (IPDA) [8,9].

For multi-target tracking in clutter with FTD, joint integrated PDA (JIPDA) [10] and joint ITS (JITS) [11] with PTE have been proposed for autonomous target tracking. JIPDA and JITS enumerate all the FJEs as JPDA and evaluate the data association probabilities and the PTE of each track. They become impractical for even a small number of closely located targets in heavily cluttered environments due to intractable number of all the FJEs for the tracking environments. To alleviate the computational burden of the multi-target data association algorithms, linear multi-target IPDA (LMIPDA) [12], linear multi-target ITS [12], and iterative JIPDA (iJIPDA) [13,14] along with efficient implementations have recently been proposed. Several deterministic approximation approaches such as suboptimal JPDA have been proposed in [15,16]. The multiple hypothesis tracking (MHT) filters [17,18] employ multi-scan data association schemes to maintain a set of measurement history hypotheses with high track scores. There are many versions of the MHT filter. Most of them can be grouped into two classes: the track-oriented MHT [18] and the measurement-oriented MHT [17]. The original random finite set (RFS) approach [2,3] to multi-target filtering does not require track-to-measurement association. However, it lacks track management functions for target tracking such as labeling and track scoring that are important in practice. Recently the RFS approaches become more practical as they are equipped with tools for track scoring, labeling and data association [19]. Markov chain Monte Carlo (MCMC) data association [20] is a stochastic method recently proposed for solving data association problems in multi-target tracking. It uses the Metropolis-Hastings algorithm to generate the FJEs. The MH algorithm is known to be an MCMC method, in which the parameters of interest or samples follow a proposed distribution to determine moving to another state or staying at the current state according to an acceptance probability. The move to a new FJE is determined by the acceptance probability which is based on the ratio of the probabilities of old and new events. As shown in the simulation experiments of [20], this MCMC data association method should generate about 10,000 burn-in samples and large MCMC samples are needed for calculation of the multi-target data association probabilities at every scan. Regarding real-time applications, this method may not be practical even though it is flexible and executable in polynomial time.

In this paper, we present a new data association algorithm that uses a Markov chain [21] to approximate the probabilities of the FJEs of JIPDA. It is called a Markov chain–based JIPDA (MCJIPDA) filter for multi-target tracking in cluttered environments. The MCJIPDA algorithm does not utilize a Monte-Carlo technique and is different from the MCMC method of [20]. The proposed method sequentially generates a Markov chain for each cluster target based on the transition probability matrix of the Markov chain model developed from IPDA for single-target tracking, which can be calculated without imposing additional computation load. The Markov chain sequences for all tracks are used to evaluate the data association probabilities of the FJEs. The number of FJEs is predetermined and the FJEs are generated from the transition probabilities based on IPDA for single target tracking at first, and later they are adjusted for the multi-target tracking environment in clutter. It is shown by simulation studies that a few hundred measurement states for each track generated by the proposed Markov chain are enough to compute the posterior data association probabilities and to maintain performance similar to that of JIPDA. This makes a big difference between the proposed algorithm and the MCMC method of [20]. Simulation studies also show that the execution time is substantially reduced compared to that of JIPDA. 

The rest of this paper is organized as follows. Section 2 revisits JIPDA for a brief introduction. The Markov chain data association algorithm for joint integrated target tracking is described in Section 3. Section 4 shows the performance of the MCJIPDA via simulations, followed by the concluding remarks in Section 5.

## 2. Target Tracking with JIPDA

This section provides an overview of JIPDA with models of target dynamics and sensor measurements. It also introduces a reformulation of the posterior probabilities of the FJEs for JIPDA to aid understanding how the sequences of the Markov chain for each target can be used to represent the FJEs. In this paper, we use superscript τ to denote a track, and a true target that track τ follows.

### 2.1. Mathematical Models

Consider a linear dynamic model of target τ described by

(1) $$x_{k+1}^{\tau }=Fx_{k}^{\tau }+\nu_{k},x_{k}^{\tau }\in R^{n_{x}}$$

where $x_{k}^{\tau }$ is the target state vector at time *k*, F is the transition matrix, and $\nu_{k}$ is a zero-mean, white Gaussian noise sequence with known variance $Q_{k}$.

Let $z_{k}^{\tau }={\left\{z_{k,i}^{\tau }\right\}}_{i=1}^{m_{k}^{\tau }}$ denote the set of validated measurements at time *k* for target τ, $z_{k,i}^{\tau }$ denotes the *i* th measurement of $z_{k}^{\tau }$, and $m_{k}^{\tau }$ denotes the number of measurements received at scan *k* in the validation gate of track τ that follows target τ. Each target can generate at most one detection per each *k* with the probability of detection $P_{D}^{\;\tau }$.

Then, the set of measurements collected up to time *k* from the entire surveillance region is denoted as

(2) $$Z^{k}=\{z_{k},\;Z^{k-1}\}$$

and $z_{k}$ contains the measurements of all the targets as well as the clutter measurements in the surveillance region at scan k and its cardinality is denoted as $m_{k}$. The number of clutter measurements is assumed to follow a Poisson distribution, and they are uniformly distributed over the entire surveillance region with the clutter measurement density $\rho_{k}$.

The measurement model from a sensor for target τ is given by

(3) $$z_{k,i}^{\tau }=\;Hx_{k}^{\;\tau }+w_{k},\;z_{k,i}^{\tau }\in R^{n_{z}}$$

where H is the measurement matrix and $w_{k}$ is a white, zero-mean Gaussian measurement noise sequence with known variance $R_{k}$.

### 2.2. JIPDA

Tracks may be initiated from target or clutter measurements. True tracks should be confirmed fast and kept confirmed, while false tracks should be terminated effectively through a proper track management method. JIPDA utilizes the PTE for track management.

The event that the target exists at time *k* is denoted by $\chi_{k}$. Then, the PTE at time *k* conditioned on $Z^{k}$ is denoted by $P(\chi_{k}|Z^{k})$, and the probability that the target does not exist satisfies

(4) $$P({\bar{\chi }}_{k}|Z^{k})=1-P(\chi_{k}|Z^{k})$$

The Markov chain-one model [8] for the propagation of the PTE for target τ is given by

(5) $$P(\chi_{k}^{\tau }|Z^{k-1})=\Delta_{11}P(\chi_{k-1}^{\tau }|Z^{k-1})+\Delta_{21}P({\bar{\chi }}_{k-1}^{\tau }|Z^{k-1})$$

where the transition probabilities are defined as

(6) $$\Delta_{11}=P(\chi_{k}^{\tau }|\chi_{k-1}^{\tau })$$

(7) $$\Delta_{21}=P(\chi_{k}^{\tau }|{\bar{\chi }}_{k-1}^{\tau })$$

The *a posteriori* probability density function (pdf) of the target state is given by

(8) $$p(x_{k}^{\;\tau },\chi_{k}^{\tau }|Z^{k})=p(x_{k}^{\;\tau }|\chi_{k}^{\tau },Z^{k})P(\chi_{k}^{\tau }|Z^{k})$$

The estimated probability density function (pdf) of the target state is conditioned on $\chi_{k}^{\tau }$. This pdf can be divided into the sum of the data association probabilities for the set of measurements by using the total probability theorem, which is given by

(9) $$p(x_{k}^{\;\tau }|\chi_{k}^{\tau },Z^{k})=\sum_{i=0}^{m_{k}}p(x_{k}^{\;\tau }|\chi_{k,i}^{\tau },\chi_{k}^{\tau },Z^{k})\beta_{i}^{\tau }$$

where $\chi_{k,i}^{\tau }$ is the hypothesis that the ith measurement of $z_{k}$ is the measurement of target τ (for i = 0, target τ is not detected) and the data association probability $\beta_{i}^{\;\tau }$ can be expressed as

(10) $$\beta_{i}^{\tau }=P(\chi_{k,i}^{\tau }|\chi_{k}^{\tau },Z^{k})\;\;=\frac{P(\chi_{k,i}^{\tau },\chi_{k}^{\tau }|Z^{k})}{P(\chi_{k}^{\tau }|Z^{k})}$$

A feasible joint event (FJE) is one possible mapping of the measurements to the tracks that follow targets. For each joint event, it is assumed that each track can be assigned to zero or one of the measurements which falls in the validation gate of the track, and each measurement can be allocated to zero or one of the tracks in order to be a FJE. Therefore, the FJE condition implies that no two tracks in a FJE share the same measurement.

Let $\kappa_{j}$ and K denote the *j*th FJE and the number of all the FJEs for data association at time *k*, respectively. Then, the sum of the a posteriori probabilities of all the FJEs satisfies

(11) $$\sum_{j=1}^{Κ}P\{\kappa_{j}|Z^{k}\}=1$$

The data association probabilities of track τ are obtained by summing over all the probabilities of FJEs that contain track τ and the measurement of interest. Denote by Ξ(τ,i) the set of FJEs in which track τ is allocated to measurement i (0 means no measurement allocation), we have

(12) $$P(\chi_{k,0}^{\tau }|Z^{k})=\sum_{e\in \Xi (\tau ,0)}P\{\kappa_{e}|Z^{k}\}$$

(13) $$P(\chi_{k,0}^{\tau },\chi_{k}^{\tau }|Z^{k})=\frac{\left(1-P_{D}^{\tau }P_{G}^{\tau })P(\chi_{k}^{\tau }|Z^{k-1}\right)}{1-P_{D}^{\tau }P_{G}^{\tau }P(\chi_{k}^{\tau }|Z^{k-1})}P(\chi_{k,0}^{\tau }|Z^{k})$$

(14) $$P(\chi_{k,i}^{\tau },\chi_{k}^{\tau }|Z^{k})=\sum_{e\in \Xi (\tau ,i)}P\{\kappa_{e}|Z^{k}\}$$

where $P_{G}^{\tau }$ is the gating probability of track τ.

The a posteriori PTE for track τ in JIPDA is obtained from the sum of the joint probabilities by

(15) $$P(\chi_{k}^{\tau }|Z^{k})=\sum_{i=0}^{m_{k}}p(\chi_{k,i}^{\tau },\chi_{k}^{\tau }|Z^{k})$$

Let $p_{k,i}^{\tau }$ denote the truncated measurement likelihood function of track τ for measurement $z_{k,i}^{\tau }\in z_{k}$ in the validation gate $V_{k}^{\tau }$ of track τ,

(16) $$p_{k,i}^{\tau }=\{\begin{array}{cc} \frac{1}{P_{G}^{\tau }}p(z_{k,i}^{\tau }|Z^{k-1}), & z_{k,i}^{\tau }\in V_{k}^{\tau }\\ 0, & z_{k,i}^{\tau }\notin V_{k}^{\tau } \end{array}$$

Now, the a posteriori probability of FJE $\kappa_{j}$ in JIPDA [10] is defined. Denote by $T_{0}^{j}$ and $T_{1}^{j}$ the set of tracks allocated with no measurements and the set of tracks allocated with one measurement for the joint event j in Equation (11), respectively. The a posteriori probability of FJE $\kappa_{j}$ is defined by

(17) $$P\{\kappa_{j}|Z^{k}\}=C^{-1}\underset{\tau \in T_{0}^{j}}{\Pi }(1-P_{D}^{\tau }P_{G}^{\tau }P\{\chi_{k}^{\tau }|Z^{k-1}\})\underset{\tau \in T_{1}^{j}}{\Pi }(P_{D}^{\tau }P_{G}^{\tau }p_{k,m(\tau ,j)}^{\tau }\rho_{k}^{-1}P\{\chi_{k}^{\tau }|Z^{k-1}\})$$

where m(τ,j) is the index of the measurement allocated to track τ in FJE $\kappa_{j}$, $p_{k,m(\tau ,j)}^{\tau }$ can be obtained by replacing the subscript i with m(τ,j) in Equation (16), $\rho_{k}$ is the clutter measurement density, and the normalization constant *C* is calculated from Equation (11).

In fact, tracks are partitioned into clusters [10]. A cluster is a set of tracks and the measurements these tracks select. In other words, the tracks not belonging to the cluster do not share any of the cluster measurements. The purpose of clustering is to minimize the number of all the FJEs by limiting the numbers of tracks and measurements inside a cluster.

**Figure 1:**
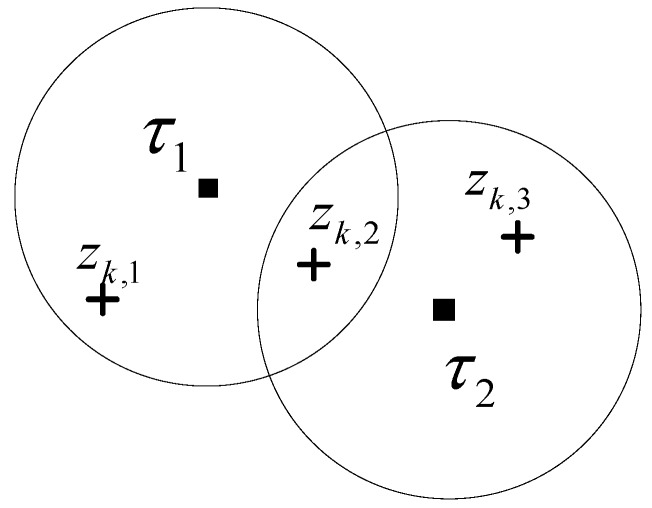
An example of a cluster for two tracks and three measurement.

The following is an example to illustrate the set of all the FJEs of JIPDA and the a posteriori probability calculation for the set. Consider the two-dimensional multi-target tracking situation depicted in Figure 1. There are two cluster tracks, labeled $\tau_{1}$ and $\tau_{2}$, and three measurements $z_{k,1}$ to $z_{k,3}$ in the cluster. For this cluster, the total number of FJEs is 8.

Each track is assigned to zero or one measurement, and each measurement is allocated to zero or one track. Two FJEs are different if at least one track-to-measurement assignment is different. All the FJEs for the cluster shown in Figure 1 are listed in Table 1. Note that we use *j* to denote the *j*-th FJE in Table 1.

Let T denote the total number of tracks in a cluster. Let $m_{k}^{C}$ and $m_{k}^{\tau }$ denote the total number of measurements in the cluster and the number of measurements in the validation gate of track τ in the cluster, respectively. The set of T tracks in the cluster, and the sets of $m_{k}^{C}$ and $m_{k}^{\tau }$ measurements are defined by

(18) $$\left\{\tau \}=\{\tau_{1},\;\tau_{2},\;\cdots ,\;\tau_{T}\right\}$$

(19) $$\left\{z_{k}^{\tau }\}=\{z_{k,m\left(\tau ,j\right)},\;\tau \in T_{1}^{j}\right\}$$

(20) $$\left\{z_{k}^{C}\}=\{z_{k,1},\;z_{k,2},\;\cdots ,\;z_{k,m_{k}^{C}}\right\}\;\mathrm{and}\;\left\{z_{k}^{\tau }\}\subset \{z_{k}^{C}\right\}$$

The number of unique assignments of $m_{k}^{C}$ measurements to T tracks, assuming that all tracks select all measurements satisfies [10]

(21) $$T!\sum_{\tau =0}^{T}\frac{1}{\tau !}\left(\begin{array}{c} m_{k}^{C}\\ T-\tau \end{array}\right)\geq (m_{k}^{C}+1)T!\;if\;m_{k}^{C}\geq T\geq 1$$

(22) $$m_{k}^{C}!\sum_{m=0}^{m_{k}^{C}}\frac{1}{m!}\left(\begin{array}{c} T\\ m_{k}^{C}-m \end{array}\right)\geq (T+1)m_{k}^{C}!\;if\;T\geq m_{k}^{C}\geq 1$$

The number of all the FJEs depends only on the number of measurements, the number of tracks, and the measurements.

Since $T_{0}^{j}$ and $T_{1}^{j}$ in Equation (17) are mutually exclusive and exhaustive in the set {τ} of cluster tracks,

(23) $$\{\tau \}=T_{0}^{j}\cup T_{1}^{j}$$

The tracks in $T_{0}^{j}$ are assigned to non-detection and the tracks in $T_{1}^{j}$ are assigned to one of the cluster measurements that is not shared by other tracks in $T_{1}^{j}$. The a posteriori probability $\left(1-P_{D}^{\tau }P_{G}^{\tau }P\{\chi_{k}^{\tau }|Z^{k-1}\}\right)$ is assigned to the tracks in $T_{0}^{j}$ and the a posteriori probability $\left(P_{D}^{\tau }P_{G}^{\tau }p_{k}^{\tau ,j}\rho_{k}^{-1}P\{\chi_{k}^{\tau }|Z^{k-1}\}\right)$ is assigned to the tracks in $T_{1}^{j}$.

Therefore, the a posteriori probability of FJE $\kappa_{j}$ can be expressed by

(24) $$P\{\kappa_{j}|Z^{k}\}=C^{-1}\prod_{\tau =\tau_{1}}^{\tau_{T}}f_{j}^{\tau }(\theta_{j}^{\tau }=z_{k,m(\tau ,j)})$$

(25) $$f_{j}^{\tau }(\theta_{j}^{\tau }=z_{k,m(\tau ,j)})=\{\begin{array}{c} 1-P_{D}^{\tau }P_{G}^{\tau }P\{\chi_{k}^{\tau }|Z^{k-1}\},\;z_{k,m(\tau ,j)}=\phi \\ P_{D}^{\tau }P_{G}^{\tau }p_{k,m}^{\tau }\rho_{k}^{-1}P\{\chi_{k}^{\tau }|Z^{k-1}\},\;z_{k,m(\tau ,j)}\;\in \{z_{k}^{\tau }\} \end{array}$$

where $\theta_{j}^{\;\tau }$ denotes the measurement state of measurement allocated to track τ in the FJE $\kappa_{j}$, and the measurement state can be no detection or a member of $\left\{z_{k}^{\tau }\right\}$, as described in Equation (25). 

If we denote that the number of all FJEs for {τ} and $\left\{z_{k}^{C}\right\}$ is K, then the following T × K matrix of which the element represents the a posteriori probability of track τ, $f_{j}^{\tau }(\theta_{j}^{\tau }=z_{k,m(\tau ,j)})$ of FJE $\kappa_{j}$. The matrix is called the a posteriori probability matrix of FJEs (PMFJE) and is denoted by $P^{JM}$ such as

(26) $$P^{JM}={\left[\begin{array}{cccc} f_{1}^{\tau_{1}}(\theta_{1}^{\tau_{1}}) & f_{2}^{\tau_{1}}(\theta_{2}^{\tau_{1}}) & \cdots & f_{Κ}^{\tau_{1}}(\theta_{Κ}^{\tau_{1}})\\ f_{1}^{\tau_{2}}(\theta_{1}^{\tau_{2}}) & f_{2}^{\tau_{2}}(\theta_{2}^{\tau_{2}}) & \cdots & f_{Κ}^{\tau_{2}}(\theta_{Κ}^{\tau_{2}})\\ \cdots & \cdots & \cdots & \cdots \\ f_{1}^{\tau_{T}}(\theta_{1}^{\tau_{T}}) & f_{2}^{\tau_{T}}(\theta_{2}^{\tau_{T}}) & \cdots & f_{Κ}^{\tau_{T}}(\theta_{Κ}^{\tau_{T}}) \end{array}\right]}_{T\times Κ}$$

where the rows represent tracks and the columns represent FJEs. The (i, j)th element of $P^{JM}$ represents the a posteriori probability of track to measurement association for track and the measurement with state $\theta_{j}^{\tau_{i}}$ for FJE $\kappa_{j}$. The a posteriori probability of FJE $\kappa_{j}$ is calculated by multiplying all the elements in the column *j* of $P^{JM}$ such as

(27) $$P\{\kappa_{j}|Z^{k}\}=C^{-1}\prod_{i=1}^{T}P_{ij}^{JM}=C^{-1}\prod_{i=1}^{T}f_{j}^{\tau_{i}}(\theta_{j}^{\tau_{i}})\;$$

where $P_{ij}^{JM}$ is an element in the ith row and the jth column of $P^{JM}$. Each column *j* of $P^{JM}$ represents the collection of elements in $P\{\kappa_{j}|Z^{k}\}$ of FJE $\kappa_{j}$. Any two measurement states $\theta_{j}^{\tau_{l}}$ and $\theta_{j}^{\tau_{n}}$ that are assigned to track $\tau_{l}$ and $\tau_{n}$, respectively, in FJE $\kappa_{j}$ should be different according to the FJE condition described in Section 2.1, i.e., $\theta_{j}^{\tau_{l}}\neq \theta_{j}^{\tau_{n}}$ if l≠n. The PMFJE for the cluster tracks shown in Figure 1 can be obtained as follows:

(28) $$P^{JM}={\left[\begin{array}{cccc} \begin{array}{cccc} \begin{array}{cc} \begin{array}{c} f_{1}^{\tau_{1}}(\phi )\\ f_{1}^{\tau_{2}}(\phi ) \end{array} & \begin{array}{c} f_{2}^{\tau_{1}}(\phi )\\ f_{2}^{\tau_{2}}(z_{k,2}) \end{array} \end{array} & \begin{array}{c} f_{3}^{\tau_{1}}(\phi )\\ f_{3}^{\tau_{2}}(z_{k,3}) \end{array} & \begin{array}{c} f_{4}^{\tau_{1}}(z_{k,1})\\ f_{4}^{\tau_{2}}(\phi ) \end{array} & \begin{array}{c} f_{5}^{\tau_{1}}(z_{k,1})\\ f_{5}^{\tau_{2}}(z_{k,2}) \end{array} \end{array} & \begin{array}{c} f_{6}^{\tau_{1}}(z_{k,1})\\ f_{6}^{\tau_{2}}(z_{k,3}) \end{array} & \begin{array}{c} f_{7}^{\tau_{1}}(z_{k,2})\\ f_{7}^{\tau_{2}}(\phi ) \end{array} & \begin{array}{c} f_{8}^{\tau_{1}}(z_{k,2})\\ f_{8}^{\tau_{2}}(z_{k,3}) \end{array} \end{array}\right]}_{2\times 8}$$

## 3. Markov Chain Based JIPDA (MCJIPDA)

The number of feasible joint events increases combinatorially with the number of measurements and the number of tracks involved, as shown in Equations (21) and (22). For tracking closely located multiple targets in heavily cluttered environments, K, the total number of all the FJEs, becomes too large for the association probability computation to be feasibly handled. This is the main reason why JPDA or JIPDA cannot be applied in real-time applications for these environments. Since the computational resource involved in two consecutive scans can vary significantly depending on the number of all the FJES for the tracking environment. It is hard to predict in advance. However, the tracking algorithms must be executed in a predictable cycle for real-time applications. Therefore, the algorithms with the reduced number FJEs are needed. The reduced size PMFJE should represent the significant joint events for data association effectively while neglecting most of insignificant joint events to approximate the data association probabilities and thus to maintain similar performance to JIPDA.

An approximated version of PMFJE is determined by the Markov chain approach in this paper by generating a T × N matrix, with N much smaller than K, using the Markov chain approach. In this paper, the proposed Markov chain data association algorithm utilizes a Markov chain to generate the FJEs. For the PMFJE, $\theta_{j}^{\;\tau }$ of $f_{j}^{\;\tau }$ in every row of $P^{JM}$ for each track τ is generated sequentially according to the Markov chain property.

A Markov chain is a sequential stochastic process. Assume that $\theta_{n}$ has state $y_{n}$ at step *n* satisfying the Markov property

(29) $$P(\theta_{n+1}=y_{n+1}|\theta_{n}=y_{n},\;\theta_{n-1}=y_{n-1},\;\ldots \;\theta_{1}=y_{1})=P(\theta_{n+1}=y_{n+1}|\theta_{n}=y_{n})$$

then the probability of moving to the next state at step *n*+1 depends on the present state $y_{n}$ but not on the past state history. By utilizing the Markov chain property of Equation (29), the state generation becomes computationally efficient as one does not need to store the entire past state histories but only the current states to generate the next states. A Markov chain process with the Metropolis-Hasting algorithm is presents for generating FJEs in [20]. A Markov chain process with randomized transition probabilities instead of deterministic values to improve modelling of stroke disease is proposed in [22]. This approach is known to be useful for decision making with real medical data.

A direct consequence of the Markov property is that the Markov chain can be generated sequentially ($\theta_{1},\;\theta_{2},\;\cdots$). For a finite discrete state set, a Markov chain can be represented by a transition probability matrix π of which the element denoted by $\pi_{uv}$ represents the transition probability from state *u* to state *v* in one step. The transition probability $\pi_{uv}$ of a homogeneous Markov chain is given by

(30) $$\pi_{uv}=\pi (\theta_{n+1}=v|\theta_{n}=u)$$

and the transition probabilities satisfy

(31) $$\sum_{v}\pi_{uv}=1$$

for every *u*. Note that *u* and *v* belong to the same measurement state set.

**Figure 2:**
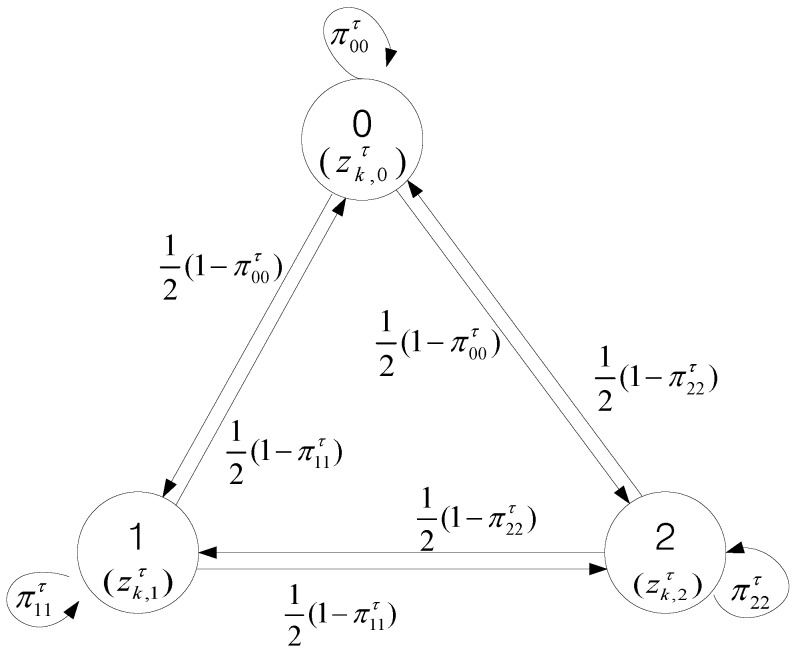
An example of a three-state Markov chain for track τ with two measurements.

To obtain the reduced size FJEs for data association with the Markov chain in this paper, the states of the Markov chain are defined as elements of the set $\phi \cup \left\{z_{k}^{\tau_{i}}\right\}$ for every track in {τ}. The move from $\theta_{n-1}^{\tau_{i}}=u$ to $\theta_{n}^{\tau_{i}}=v$ is accepted with the transition probability $\pi_{uv}^{\tau_{i}}$, for j=1,2, …, T. A sequence ${\left\{\theta_{n}^{\tau_{i}}\right\}}_{1}^{N}$ with *N* elements is generated from the set with the transition probability matrix developed from IPDA for single target tracking in clutter. We call it a Markov chain measurement allocation sequence (MCMAS). The MCMAS is applied for other tracks to form a FJE. Design of the Markov chain with the transition probabilities developed from IPDA is illustrated in Section 4. If $\theta_{n}^{\tau_{i+1}}=v\neq \phi$ is the same as $\theta_{n}^{\tau_{i}}=v$ that is the measurement state selected by another track, then regenerate $\theta_{n}^{\tau_{i+1}}$ until it avoids v to ensure the FJE condition of multi-target tracking. The procedure can be illustrated with an example shown in Figure 1, each MCMAS of length N=5 for each track is generated as following. For track $\tau_{1}$, the first MCMAS is generated from a measurement set $\left\{0,\;z_{k,1},\;z_{k,2}\right\}$ where ‘0’ means no assignment case. By the same way, the second MCMAS of track $\tau_{2}$ is generated from a measurement set $\left\{0,\;z_{k,2},\;z_{k,3}\right\}$. For example, the first MCMAS in length 5 can be ${\left\{\theta_{n}^{\text{​}\;1}\right\}}_{n=1}^{5}=\{z_{k,1},\;z_{k,1},\;0,\;z_{k,2},\;z_{k,1}\}$, which is generated from the Markov chain random process, as shown in Figure 2. The second MCMAS can be obtained as ${\left\{\theta_{n}^{\;2}\right\}}_{n=1}^{5}=\{z_{k,2},\;z_{k,3},\;0,\;z_{k,2},\;0\}$. The next is to check the measurement-to-track assignment from the MCMAS for the FJE condition for $\kappa_{n}$, which is represented by $\left\{\theta_{n}^{\;1},\;\theta_{n}^{\;2}\right\}$. In this case, $\kappa_{1}=\{z_{k,1},\;z_{k,2}\}$, $\kappa_{2}=\{z_{k,1},\;z_{k,3}\}$, $\kappa_{3}=\{0,\;0\}$, $\kappa_{4}=\{z_{k,2},\;z_{k,2}\}$ and $\kappa_{5}=\{z_{k,1},\;0\}$. Among them $\kappa_{4}=\{z_{k,\;2},\;z_{k,\;2}\}$ violates the FJE condition that implies no two tracks share the same measurement. In the above example, $\theta_{4}^{\;2}$ of track $\tau_{2}$ is the same as $\theta_{4}^{\;1}$ of track $\tau_{1}$, so $\theta_{4}^{\;2}$ of track $\tau_{2}$ is regenerated to avoid $\theta_{4}^{\;2}$ and to ensure the FJE condition. This can be done by checking the assigned elements in the same column of $P^{JM}$ before moving to the next column. 

The approximated PMFJE, $P^{JM}$ is completed by using the probability weight $f_{n}^{\tau }(\theta_{n}^{\tau }=z_{k,m(\tau ,n)})$ defined in Equation (25), and the *a posteriori* probability of joint event $\kappa_{n}$ is obtained by replacing index j with the index n in Equation (27). The normalization constant for the approximated $P^{JM}$ is obtained from $\sum_{n=1}^{N}P\{\kappa_{n}|Z^{k}\}=1$.

After the Markov chain track sequences, ${\left\{\theta_{n}^{\tau }\right\}}_{1}^{N}$ for T cluster tracks is obtained, the approximated PMFJE $P^{JM}$ is completed. From the approximated $P^{JM}$, the track to measurement association probabilities $\beta_{o}^{\;\tau }$ and $\beta_{i}^{\;\tau }$ are obtained from Equation (10) and the a posteriori PTE for track τ in {τ} is obtained from Equation (15).

## 4. Design of Transition Probabilities for MCJIPDA

The data association probabilities of IPDA are utilized for developing the transition probabilities of the proposed Markov chain. Consider $m_{k}^{\tau }$ validated measurements for a track τ among the cluster tracks of a multi-target tracking situation. Denote by $\phi \cup \{z_{k}^{\tau }\}=\{z_{k,0},\;z_{k,1},\;\cdots ,\;z_{k,m_{k}^{\tau }}\}$ the joint set of measurements in the validation gate, where $z_{k,0}$ represents the case that no measurement is allocated to track τ. The transition matrix $\pi^{\tau }$ of track τ for the purpose of generating the sequence of length N to represent the FJEs can be defined as

(32) $$\pi_{\xi \eta }^{\tau }=\pi^{\tau }\left(\theta_{n+1}^{\tau }=z_{k,\eta }|\theta_{n}^{\tau }=z_{k,\xi }\right)$$

where

(33) $$\pi_{\xi \xi }^{\tau }=\{\begin{array}{c} \frac{1-P_{D}^{\tau }P_{G}^{\tau }P\{\chi_{k}^{\tau }|Z^{k-1}\}}{\lambda_{k}},\;\xi =0\\ \frac{P_{D}^{\tau }P_{G}^{\tau }p_{k,\xi }^{\tau }\rho_{k}^{-1}P\{\chi_{k}^{\tau }|Z^{k-1}\}}{\lambda_{k}},\;\xi =1,2,\ldots m_{k}^{\tau } \end{array}$$

which is based on the posterior probability of joint event in Equation (25). Besides, it is assumed that the state of measurement assignment changes to another state at the next step with the transition probability given by

(34) $$\pi_{\xi \eta }^{\tau }=\{\begin{array}{c} \frac{1}{m_{k}^{\tau }}\left(1-\frac{1-P_{D}^{\tau }P_{G}^{\tau }P\{\chi_{k}^{\tau }|Z^{k-1}\}}{\lambda_{k}}\right),\;\xi =0,\xi \neq \eta \\ \frac{1}{m_{k}^{\tau }}\left(1-\frac{P_{D}^{\tau }P_{G}^{\tau }p_{k,\xi }^{\tau }\rho_{k}^{-1}P\{\chi_{k}^{\tau }|Z^{k-1}\}}{\lambda_{k}}\right),\;\xi \neq 0,\xi \neq \eta \end{array}$$

The measurement likelihood ratio $\lambda_{k}$ in the diagonal terms of the transition matrix is given by [8]

(35) $$\lambda_{k}=1-P_{D}^{\tau }P_{G}^{\tau }P\{\chi_{k}^{\tau }|Z^{k-1}\}+\sum_{\xi =1}^{m_{k}^{\tau }}P_{D}^{\tau }P_{G}^{\tau }p_{k,\xi }^{\tau }\rho_{k}^{-1}P\{\chi_{k}^{\tau }|Z^{k-1}\}$$

Furthermore, $\pi_{\xi \eta }^{\tau }$ is defined to sum to one for every ξ from the property of Markov chain

(36) $$\sum_{\eta =0}^{m_{k}^{\tau }}\pi_{\xi \eta }=1$$

which implies that the non-diagonal elements in the same row of the transition matrix have the same likelihood for transition. For example, the Markov chain for track τ with the set of measurements $\left\{z_{k}^{\tau }\}=\{z_{k,1},z_{k,2}\right\}$ is depicted in Figure 2. In this example, the transition probability matrix becomes

(37) $$\pi^{\;\tau }=\left[\begin{array}{ccc} \pi_{00}^{\tau } & \pi_{01}^{\tau } & \pi_{02}^{\tau }\\ \pi_{10}^{\tau } & \pi_{11}^{\tau } & \pi_{12}^{\tau }\\ \pi_{20}^{\tau } & \pi_{21}^{\tau } & \pi_{22}^{\tau } \end{array}\right]=\left[\begin{array}{ccc} \frac{1-P_{D}^{\tau }P_{G}^{\tau }P\{\chi_{k}^{\tau }|Z^{k-1}\}}{\lambda_{k}} & \frac{1}{2}(1-\pi_{00}^{\tau }) & \frac{1}{2}(1-\pi_{00}^{\tau })\\ \frac{1}{2}(1-\pi_{11}^{\tau }) & \frac{P_{D}^{\tau }P_{G}^{\tau }p_{k,1}^{\tau }\rho_{k}^{-1}P\{\chi_{k}^{\tau }|Z^{k-1}\}}{\lambda_{k}} & \frac{1}{2}(1-\pi_{11}^{\tau })\\ \frac{1}{2}(1-\pi_{22}^{\tau }) & \frac{1}{2}(1-\pi_{22}^{\tau }) & \frac{P_{D}^{\tau }P_{G}^{\tau }p_{k,2}^{\tau }\rho_{k}^{-1}P\{\chi_{k}^{\tau }|Z^{k-1}\}}{\lambda_{k}} \end{array}\right]$$

If the (*n*−1)-th assignment of MCMAS for track τ is $\theta_{n-1}^{\tau }=z_{k,1}$, the *n*-th element of MCMAS is generated according to the second row of the transition probability matrix in Equation (38) and a uniform random number $C_{Unif}$ in the interval [0, 1]. If the random number satisfies $0\leq C_{Unif}\leq \pi_{10}^{\tau }$, then $\theta_{n}^{\tau }=z_{k,0}$. If the random number satisfies $\pi_{10}^{\tau }<C_{Unif}\leq \pi_{10}^{\tau }+\pi_{11}^{\tau }$, then the measurement $z_{k,1}$ is assigned to $\theta_{n}^{\tau }$. Similarly, if $\pi_{10}^{\tau }+\pi_{11}^{\tau }<C_{Unif}\leq 1$, then $\theta_{n}^{\tau }=z_{k,2}$.

By utilizing the data association probabilities of IPDA for each track in the cluster as acceptance probabilities of the measurement state generation in the form of transition probabilities of the Markov chain, the proposed method does not need a Monte Carlo algorithm that may produce a large number of burn-in samples.

The MCMAS of length N for track τ is the key to resolve the computational risks of JIPDA. It is obvious that the FJEs of JIPDA are within the tractable range in the situations where targets are not closely located in the surveillance region. However, the target-crossing situations, the FJEs increase to prohibitively high numbers. Therefore, we propose an algorithm switch method according to the number of FJEs. If the number of FJEs is less than the length of the MCMAS predetermined for the Markov chain data association, the JIPDA algorithm is applied, and otherwise, the Markov chain data association algorithm is used and plays an important role in reducing the computational load. 

The proof of convergence of the target tracking performance of the proposed algorithm to that of the JIPDA algorithm is left for future studies. However, the convergence is shown through a series of simulations in Section 5.

## 5. Simulations

**Figure 3:**
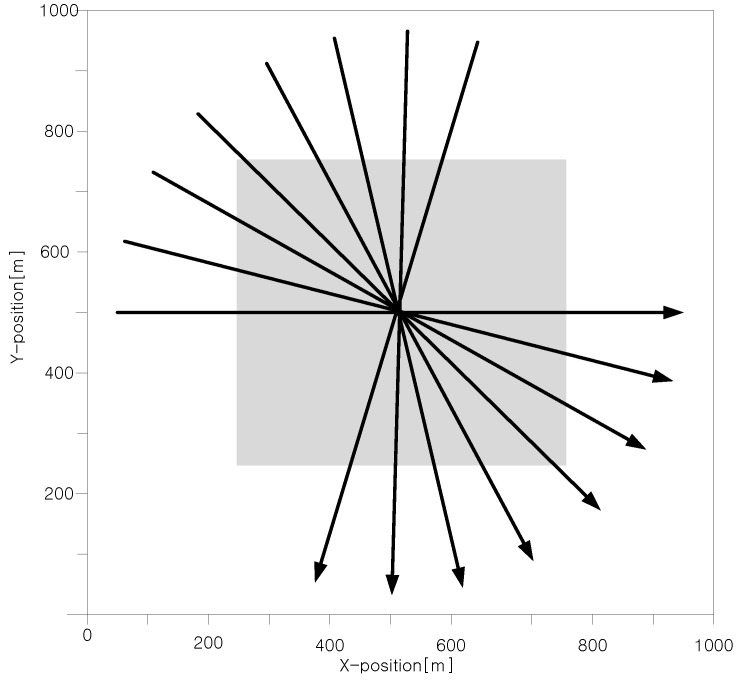
Target scenario for simulation.

The two-dimensional surveillance region considered in the simulation study is shown in Figure 3. The surveillance area is 1000 m long (*x*-axis) and 1000 m wide (*y*-axis), and the sensor scan time T=1 s. Eight targets move uniformly at a constant speed of 22.5 m/s during the time of 40 scans and cross each other at about scan 20. The clutter measurements follow a Poisson distribution with clutter densities of $\rho_{L}{/\mathrm{scan}/m}^{2}$ in the sparse clutter region and $\rho_{H}{/\mathrm{scan}/m}^{2}$ in the dense clutter region, which is 500 m long and 500 m wide as indicated by the gray area in Figure 3. Three cases for performance comparison with different detection probability $P_{D}$ and different clutter density are listed in Table 2.

The state vector $x_{k}^{\tau }$ of target τ in Equation (1) consists of the two-dimensional position and velocity in Cartesian coordinates with transition matrix

(38) $$F=\left[\begin{array}{cc} F(T) & 0_{2\times 2}\\ 0_{2\times 2} & F(T) \end{array}\right],\;F(T)=\left[\begin{array}{cc} 1 & T\\ 0 & 1 \end{array}\right]$$

where $0_{2\times 2}$ is a 2 × 2 null matrix. The variance of the process noise in Equation (1) is known to be

(39) $$Q_{k}=q\left[\begin{array}{cc} Q(T) & 0_{2\times 2}\\ 0_{2\times 2} & Q(T) \end{array}\right],\;Q(T)=\left[\begin{array}{cc} T^{4}/4 & T^{3}/2\\ T^{3}/2 & T^{2} \end{array}\right]$$

where $q=0.75\;m^{2}\;/s^{4}$. 

The measurement noise covariance matrix $R_{k}$ for the sensor is

(40) $$R_{k}=\left[\begin{array}{cc} 25 & 0\\ 0 & 25 \end{array}\right]m^{2}$$

The propagation probabilities of Markov chain-one target existence in Equation (5) are given by

(41) $$\left[\Delta_{11\;}\;\Delta_{21}]\;=[0.98\;\;\;0\right]$$

Each simulation experiment consists of 300 Monte Carlo runs. The tracks are initiated using two-point differencing initialization [10] with an initial PTE of 0.01. The tracks are confirmed if the PTE exceeds the confirmation threshold and they are eliminated if the PTE falls below the termination threshold of 0.006. 

**Figure 4:**
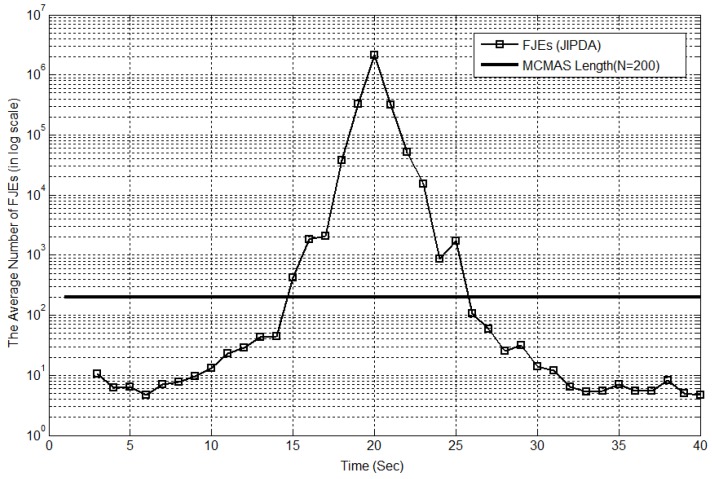
The average number of feasible joint events (FJEs) of joint integrated probabilistic data association (JIPDA) for Case #1.

Figure 4 shows the average number of all the FJEs of JIPDA for Case #1 at every scan, in which the maximum mean number of FJEs is shown to be 44,209,586 at scan 20. The scenario of this simulation corresponds to that in Figure 3. The *x*-axis indicates the scan number, and the *y*-axis represents the mean number of FJEs in a logarithmic scale obtained from 300 Monte Carlo simulation runs, and the length of the MCMAS (N = 200) is shown for comparison. The number of all the FJEs increases sharply toward the target-crossing time.

The track retention statistics to check the number of the confirmed true tracks after the target crossing at scan 20 are obtained by counting the number and identifying the track label of the confirmed true tracks at scan 15 before the target crossing and at scan 35 after the target crossing. The following statistics are accumulated:

- nCases: the total number of targets being followed by a confirmed track at scan 15;
- nOK: the number of “nCases” tracks that still follow their original tracks at scan 35;
- nSwitched: the number of “nCases” tracks that follow different targets at scan 35;
- nLost: the number of “nCases” tracks becoming false or terminated at scan 35;
- nMerge: the number of “nCases” tracks lost due to merging among “nCases tracks” at scan 35;
- nResult [CT]: the total number of targets being followed by a confirmed track at the last scan 40;
- CFT: the total number of confirmed false tracks during the entire simulation;
- CPU [sec]: the total CPU times for 300 Monte Carlo runs, in seconds, on a 3.6G Intel PC, running Windows 7, and C++ programs.

**Figure 5:**
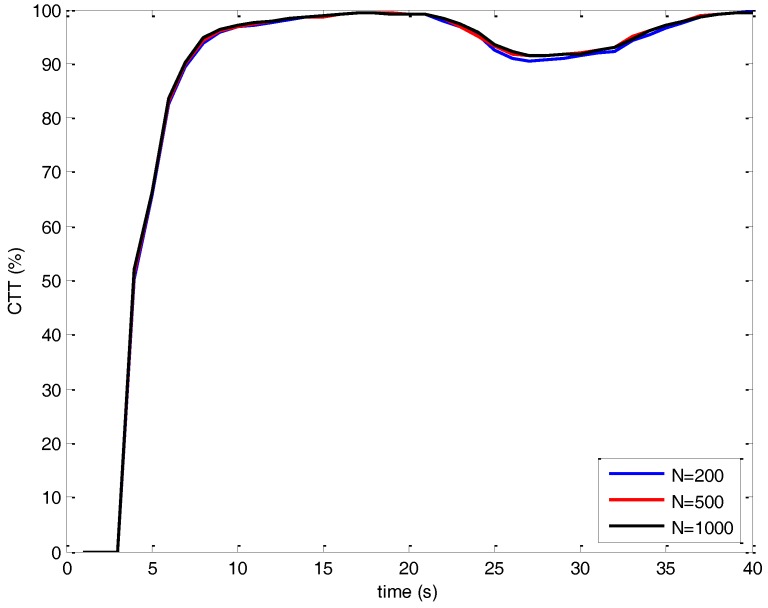
Confirmed true track (CTT) rate for different Markov Chain (MC) lengths.

To determine the MCMAS lengths, we simulate the same scenario for *N* = 200, *N* = 500 and *N* = 1000 with the proposed MCJIPDA algorithm. The confirmed true track (CTT) rates are shown in Figure 5 and track retention statistics are shown in Table 3. The CTT rate indicates that the number of CTTs versus the number of targets at each scan of the Monte Carlo runs. As the length of MCMAS increases, the performance of MCJIPDA becomes improved. However, the computational load also increases as shown in Table 3. For the 3 MCMAS lengths, the CTT rates are almost same whereas the other track retention statistics are different. Table 3 indicates that the track retention statistics of *N* = 500 show improvement compared to those with *N* = 200. However, the target retention statics are shown to be similar for *N* = 500 and *N* = 1000. To save the computational sources without deteriorating the tracking performances, *N* = 500 is selected for MCJIPDA in the simulation studies.

We compare the proposed MCJIPDA with IPDA, LMIPDA, JIPDA, and iJIPDA with level 2 in terms of the FTD performance and the track retention statistics obtained from Monte Carlo runs. The MCJIPDA algorithm is simulated by using Markov chain sequences with the length of 500 for data association at every scan when the number of cluster tracks is more than 1. As mentioned in the previous section, when the FJEs in the cluster tracks is less than N, JIPDA can be used instead of the proposed MCJIPDA. Otherwise, the MCJIPDA generates the N-length FJEs for the cluster tracks.

**Figure 6:**
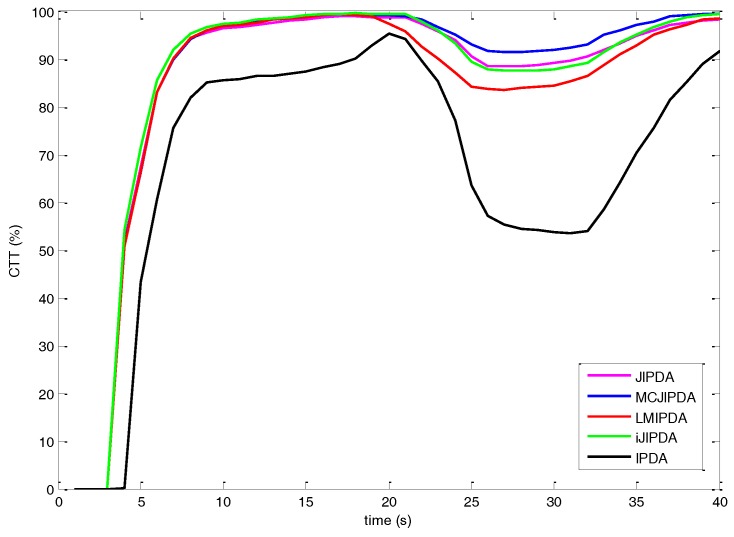
The confirmed true tracks rate for Case #1.

**Figure 7:**
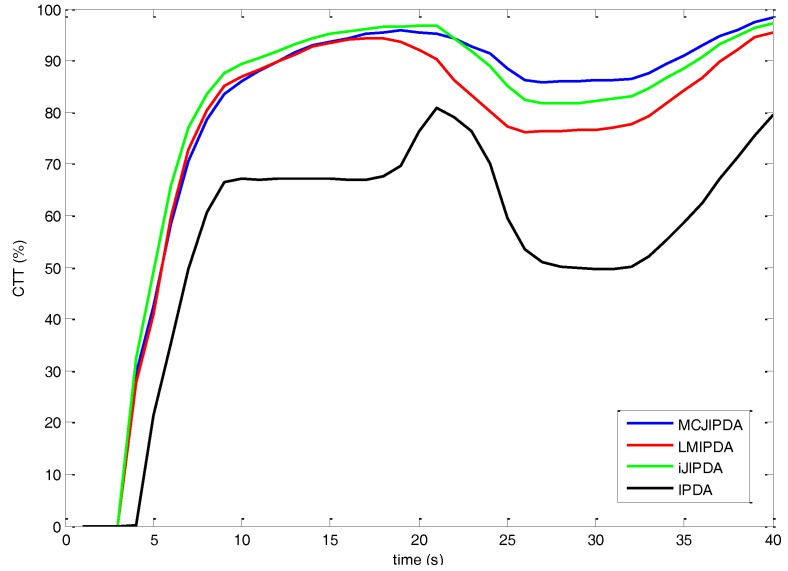
The confirmed true tracks rate for Case #2.

**Figure 8:**
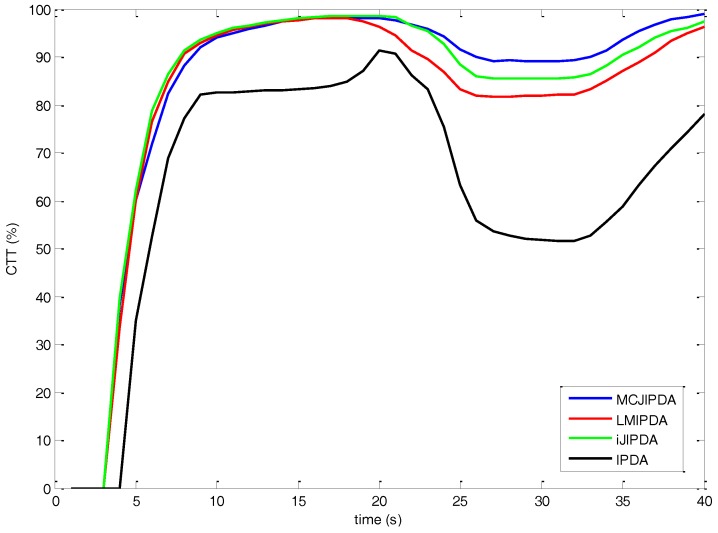
The confirmed true tracks rate for Case #3.

The FTD is shown as the CTT rate in Figure 6, Figure 7 and Figure 8. The number of confirmed false tracks (for the scenarios with different parameters listed in Table 2) for each multi-target tracking algorithm should be kept almost same for fair comparison. To achieve this, the initial PTEs of all the algorithms are set to be the same, whereas the confirmation threshold of each algorithm is adjusted within the range from 0.995 to 0.9999 to produce approximately 71 to 75 confirmed false tracks. In this scenario, the complete number of confirmed true tracks is 2400 for 300 Monte Carlo runs. As the maximum numbers of FJEs of JIPDA in Case #2 and Case #3 are over $10^{11}$. The JIPDA algorithm cannot perform tracking in real time. The simulation results of JIPDA are not shown for these two cases. 

The simulation results and the CTT rates of Case #1 are shown in Figure 6 and Table 4. As shown in Figure 6, the MCJIPDA has the best performance on the CTT rate after the target crossing. The single-target tracker, IPDA, has poor performance in this multi-target tracking scenario. The CTT rates of JIPDA and iJIPDA are almost same. In Table 4, though the MCJIPDA has a larger number of nSwitched than the other trackers, it has much smaller number of nMerged. Besides, the nResults of MCJIPDA and iJIPDA are bigger than that of IPDA, LMIPDA, and JIPDA. 

The simulation results for Case #2 are shown in Figure 7 and Table 5. Compared to Figure 6, the CTT rates of all the algorithms are sluggish and smaller in this reduced $P_{D}$ environment. As shown in Figure 7, though increase of the CTT rate of MCJIPDA is slower than those of LMIPDA and iJIPDA in the initial phase, the MCJIPDA has the best performance on the CTT rate after targets crossing. The single-target tracker, IPDA, also shows poor performance in this multi-target tracking scenario. In Table 5, though nSwitched of MCJIPDA is the biggest, but nMerged is the smallest among the algorithms in comparison. Besides, nResults of MCJIPDA is the biggest.

The simulation results for Case #3 are shown in Figure 8 and Table 6. The CTT rate for each algorithm shows similar trend shown in Figure 6 and Figure 7. Compared to Figure 6, the increase of the clutter densities affects the performance of the CTT rate, which shows slower and smaller than Case #1 for each algorithm compared to Case #1 in general. As shown in Figure 8, the increase of the CTT rate of MCJIPDA is slower than those of LMIPDA and iJIPDA, and MCJIPDA has the best performance on the CTT rate after target crossing. 

**Figure 9:**
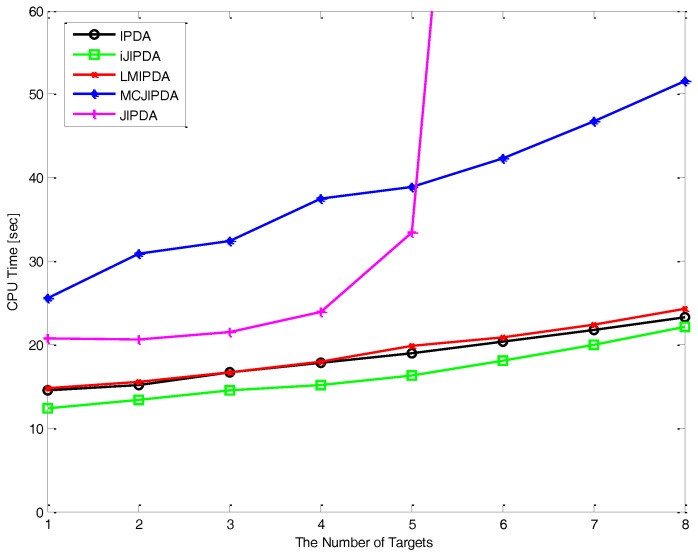
Computational costs of algorithms vary with the number of targets.

In Figure 9, it is shown that how the computational cost varies with the number of targets and the number of measurements. When the number of target varies from 1 to 8, the computational load represented by CPU time is shown for IPDA, LMIPDA, iJIPDA, JIPDA, and MCJIPDA with the length 500 of MCMAS for the tracking environment of Case #1. IPDA, LMIPDA, iJIPDA, and MCJIPDA have linearly increasing CPU time for the number of targets. JIPDA has exponentially increasing time as the number of target increases. CPU times are measured on a 3.6G Intel PC running Windows 7 and C++ programs. All the algorithms in comparison are programmed by the authors and implemented without performance optimization and parallel computation.

## 6. Conclusions

This paper presents a practical Markov chain data association algorithm for approximating the probabilities of the FJEs of JIPDA, which would otherwise incur a prohibitively heavy computational load for closely located multi-target tracking in clutter. The proposed MCJIPDA algorithm sequentially generates the measurements by a Markov chain of events for each cluster target based on the transition probabilities established from IPDA for single target tracking. The events are adjusted by event regeneration to satisfy the feasible joint event condition for multi-target tracking. The length of MCMAS for the MCJIPDA algorithm is selected through simulation studies by checking the tracking performance as well as the computation time. In the simulation studies, the proposed MCJIPDA algorithm is compared with the other existing data association methods for several simulation scenarios. The simulation results show that the MCJIPDA with 500 selected events for joint data association is comparable to JIPDA with respect to false track discrimination and target retention outcomes but with substantially less computational load. This implies that the proposed MCJIPDA algorithm provides a viable solution for multi-target tracking in clutter, especially for tracking closely located targets in dense clutter.

## Figures and Tables

**Table 1:** Joint event set of the cluster in Figure 1 (‘ϕ ’ means no allocation of measurements).

*j*	$\tau_{1}$ (=1)	$\tau_{2}$ (=2)	$P\{\kappa_{j}|Z^{k}\}$
1	ϕ	ϕ	$C^{-1}(1-P_{D}^{1}P_{G}^{1}P\{\chi_{k}^{1}|Z^{k-1}\})(1-P_{D}^{2}P_{G}^{2}P\{\chi_{k}^{2}|Z^{k-1}\})$
2	ϕ	$z_{k,m(2,2)}$ = $z_{k,2}$	$C^{-1}(1-P_{D}^{1}P_{G}^{1}P\{\chi_{k}^{1}|Z^{k-1}\})(P_{D}^{2}P_{G}^{2}p_{k,2}^{2}\rho_{k}^{-1}P\{\chi_{k}^{2}|Z^{k-1}\})$
3	ϕ	$z_{k,m(2,3)}$ = $z_{k,3}$	$C^{-1}(1-P_{D}^{1}P_{G}^{1}P\{\chi_{k}^{1}|Z^{k-1}\})(P_{D}^{2}P_{G}^{2}p_{k,3}^{2}\rho_{k}^{-1}P\{\chi_{k}^{2}|Z^{k-1}\})$
4	$z_{k,m(1,4)}$ = $z_{k,1}$	ϕ	$C^{-1}(P_{D}^{1}P_{G}^{1}p_{k,1}^{1}\rho_{k}^{-1}P\{\chi_{k}^{1}|Z^{k-1}\})(1-P_{D}^{2}P_{G}^{2}P\{\chi_{k}^{2}|Z^{k-1}\})$
5	$z_{k,m(1,5)}$ = $z_{k,1}$	$z_{k,m(2,5)}$ = $z_{k,2}$	$C^{-1}(P_{D}^{1}P_{G}^{1}p_{k,1}^{1}\rho_{k}^{-1}P\{\chi_{k}^{1}|Z^{k-1}\})(P_{D}^{2}P_{G}^{2}p_{k,2}^{2}\rho_{k}^{-1}P\{\chi_{k}^{2}|Z^{k-1}\})$
6	$z_{k,m(1,6)}$ = $z_{k,1}$	$z_{k,m(2,6)}$ = $z_{k,3}$	$C^{-1}(P_{D}^{1}P_{G}^{1}p_{k,1}^{1}\rho_{k}^{-1}P\{\chi_{k}^{1}|Z^{k-1}\})(P_{D}^{2}P_{G}^{2}p_{k,3}^{2}\rho_{k}^{-1}P\{\chi_{k}^{2}|Z^{k-1}\})$
7	$z_{k,m(1,7)}$ = $z_{k,2}$	ϕ	$C^{-1}(P_{D}^{1}P_{G}^{1}p_{k,2}^{1}\rho_{k}^{-1}P\{\chi_{k}^{1}|Z^{k-1}\})(1-P_{D}^{2}P_{G}^{2}P\{\chi_{k}^{2}|Z^{k-1}\})$
8	$z_{k,m(1,8)}$ = $z_{k,2}$	$z_{k,m(2,8)}$ = $z_{k,3}$	$C^{-1}(P_{D}^{1}P_{G}^{1}p_{k,2}^{1}\rho_{k}^{-1}P\{\chi_{k}^{1}|Z^{k-1}\})(P_{D}^{2}P_{G}^{2}p_{k,3}^{2}\rho_{k}^{-1}P\{\chi_{k}^{2}|Z^{k-1}\})$

**Table 2:** Simulation cases.

Case	$P_{D}$	$\rho_{L}$	$\rho_{H}$
#1	0.9	$10^{-5}$	$10^{-4}$
#2	0.8	$10^{-5}$	$10^{-4}$
#3	0.9	$2\times 10^{-5}$	$2\times 10^{-4}$

**Table 3:** Statistics for different MC lengths.

	MC Length	*N* = 200	*N* = 500	*N* = 1000
Statistics				
nCases		2378	2380	2379
nOK		1999	2035	2044
nSwitched		155	142	133
nLost		24	18	15
nMerged		200	185	187
nResults		2390	2385	2387
CFT		73	74	74
CPU [sec]		43.4	52.7	86.4

**Table 4:** Track retention statistics for Case #1.

Measure Items	IPDA	LMIPDA	JIPDA	iJIPDA	MCJIPDA
nCases	2121	2382	2370	2386	2380
nOK	979	1882	2039	2017	2035
nSwitched	135	105	60	64	142
nLost	7	3	5	7	18
nMerged	1000	392	266	298	185
nResult[CT]	2200	2363	2358	2386	2385
C/F Track	74	72	73	73	74
CPU[sec]	29.7	23.9	1120271	21.51	52.7

**Table 5:** Track retention statistics for Case #2.

Measure Items	IPDA	LM-IPDA	iJIPDA	MCJIPDA
nCases	1608	2256	2293	2264
nOK	693	1629	1787	1762
nSwitched	129	159	121	214
nLost	7	5	6	38
nMerged	779	463	379	250
nResult[CT]	1906	2291	2331	2359
C/F Track	73	74	71	73
CPU[sec]	28.6	25.6	21.9	53.3

**Table 6:** Track retention statistics for Case #3.

Measure Items	IPDA	LM-IPDA	iJIPDA	MCJIPDA
nCases	2002	2355	2360	2354
nOK	921	1849	1963	1984
nSwitched	105	84	56	127
nLost	9	4	7	27
nMerged	967	418	334	216
nResult[CT]	1872	2309	2336	2374
C/F Track	73	71	75	73
CPU[sec]	58.6	62.5	54.2	127.6
